# Genetic and epigenetic changes in the development of multiple colorectal cancers in the general population

**DOI:** 10.1038/sj.bjc.6602870

**Published:** 2005-11-08

**Authors:** T Watanabe, T Kanazawa, Y Kazama, J Tanaka, T Tanaka, T Tada, H Nagawa

**Affiliations:** 1 Department of Surgical Oncology, University of Tokyo Hospital, 7-3-1, Hongo, Bunkyo-ku, Tokyo 113-8655, Japan

**Sir**,

We read the interesting article by Lawes *et al* (2005) on the role of MLH1, MSH2 and MSH6 in the development of multiple colorectal cancers in the general population. They suggested that, although MSI-H is more commonly identified in those with multiple colorectal cancers, this does not commonly arise from a classical HNPCC pathway. Also, unlike HNPCC cancers, they showed the heterogeneity of the MSI status between multiple cancers within each individual in the general population. In HNPCC, about 90% of cancers show MSI. On the other hand, of the 32 patients from whom two or more cancers were analysed in Lawes’s study, eight (25%) demonstrated MSI-H in both cancers, 13 (41%) demonstrated MSI-H in one cancer and 11 (34%) failed to demonstrate any MSI-H. Although they examined only cancer lesions, we have performed a similar research for patients with multiple colorectal cancers and we further examined the MSI status in adenomas as well as cancers (Ueda *et al*, 2002). Including adenomas, we could show that the MSI status differs between tumours within each individual. Among 13 patients with multiple colorectal cancers, there was only one patient (7.7%), whose cancers and adenomas showed the same MSI status. In the rest of 12 patients, the MSI status differed between cancers and adenomas. These results confirm that neoplastic lesions in patients with multiple colon cancers in the general population do not develop in the same pathway as HNPCC. In HNPCC, not only cancers but also adenomas are known to show high frequency of MSI (Watanabe *et al*, 1996; Velayos *et al*, 2005).

Lawes *et al* also pointed out an importance of methylation of the MLH1 promoter region in the development of MSI-H cancers among patients with multiple colorectal cancers. In our analysis, in addition to MLH1, we further examined the methylation status of four more markers (p16, MINT1, MINT2, MINT 31) and classified tumours into CpG island methylator phenotype (CIMP), either CIMP-High or CIMP-Low (Tada *et al*, 2003). Methylation of hMLH1 was observed in 54% (24 out of 44) of all tumours and 78% (seven out of nine) of MSI-H tumours. CIMP-High was observed in 26% (12 out of 46) and CIMP-Low in 43% (20 out of 46) of tumours. The overall frequency of CIMP was 70% (32 out of 46) in multiple colorectal cancers, and our study demonstrated that multiple cancers show a high frequency of CIMP.

Considering that CIMP tumours develop due to epigenetic changes of various genes, our results, together with Lawes’s results, suggest that epigenetic changes play an important role in the development of multiple primary cancers in the general population.
